# Influence of presence in an inter-professional simulation training of the emergency caesarean section: a cross-sectional questionnaire study

**DOI:** 10.1007/s00404-022-06465-9

**Published:** 2022-02-26

**Authors:** Markus Flentje, Vera Hagemann, Lars Brodowski, Spiyridon Papageorgiou, Constantin von Kaisenberg, Hendrik Eismann

**Affiliations:** 1 Department of Anaesthesiology and Intensive Care Medicine, Hannover Medical School, Carl-Neuberg-Strasse 1, 30625 Hannover, Germany; 2 Faculty of Business Studies and Economics, Business Psychology, University of Bremen, Enrique-Schmidt-Strasse 1, 28359 Bremen, Germany; 3 Department of Obstetrics and Gynecology, Hannover Medical School, Carl-Neuberg-Strasse 1, 30625 Hannover, Germany

**Keywords:** Interdisciplinary collaboration, Simulation, Virtual reality, Training success

## Abstract

**Purpose:**

Emergency training using simulation is a method to increase patient safety in the delivery room. The effect of individual training concepts is critically discussed and requires evaluation. A possible influence factor of success can be the perceived reality of the participants. The objective of this study was to investigate whether the presence in a simulated emergency caesarean section improves subjective effect of the training and evaluation.

**Methods:**

In this observation study, professionals took part in simulated emergency caesarean sections to improve workflow and non-technical skills. Presence was measured by means of a validated questionnaire, effects and evaluation by means of a newly created questionnaire directly after the training. Primary outcome was a correlation between presence and assumed effect of training and evaluation.

**Results:**

106 participants (70% of course participants) answered the questionnaires. Reliability of the presence scale was good (Cronbach’s alpha 0.72). The presence correlated significantly with all evaluated items of non-technical skills and evaluation of the course. The factor “mutual support” showed a high effect size (0.639), the overall evaluation of the course (0.395) and the willingness to participate again (0.350) a medium effect. There were no differences between the professional groups.

**Conclusion:**

The presence correlates with the assumed training objectives and evaluation of the course. If training is not successful, it is one factor that needs to be improved.

## Introduction

Reducing maternal and neonatal mortality is a global effort for years [1, 2]. Meanwhile, the concept of “maternal near miss morbidity” is being pursued, which includes the period around birth in the observation period [3]. Analogous to other areas of healthcare, the working circumstances of obstetrics fulfil the framework of a high responsibility team (HRT). Death as a result of decisions made, the irreversibility of many therapeutic decisions and time pressure are some characteristics of this work environment [4]. Like in aviation settings, better training of emergencies has been a widespread recommendation [1, 2] and simulation trainings with technical and non-technical contents were implemented [5]. However, success has not always been proven and the content and structure of trainings is debated [6]. Draycott et al. testify, that training is expensive, therefore if training is done, it should be ‘effective training’. This means a reduction in mortality and morbidity and improved outcomes [7].

Evaluation of training and its impact in the patient care can be difficult, especially when it comes to identify success factors. Team composition, training location, didactics and content vary between the studies and complicate the comparability. For example, the debriefing of scenarios is recognized as the most important feature of simulation-based medical education [8] and is not always described methodically.

In this study, we focused on the relevance of perceived reality of the participant. For the description of the participant’s perceived reality, a wide range of different terms and scoring systems are used. Fiction contract [9] and immersion [10] are some examples. In our study, we used the “Presence Scale for Lab-based Microworld Research” (PLBMR), which is available in a German language validated form [11] and adapted to the health care context [12].

Even though studies found positive correlations between perceived degree of reality and learning outcomes [13, 14], we have not yet found any correlation between presence and changes in the willingness of participants to work in teams [15]. However, learning non-technical and technical skills could be different on presence.

The aim of the study was to assess the impact of presence on the subjective training value to the participant in an emergency caesarean section training. The used simulation course for this purpose was already established [16] and was successfully tested positively for transfer to everyday clinical practice [17].

The results are used, to value presence as a quality marker for simulation scenarios to integrate them into a corresponding course. Our hypotheses were: the scales of PLBMR are applicable for a German inter-professional delivery room team (a), different professional groups rate the training differently (b), the presence correlates with the subjective effect on non-technical skills (c), the presence correlates with the evaluation of the course in regard to significance for daily work (d), overall evaluation (e), and willingness to participate the training again (f).

## Methods

The study was designed as a retrospective study using survey methodology with participants from an emergency caesarean section training program (HAINS Safety simulation program). The measurements were taken immediately after the training course. The study was approved by the ethics committee of Hannover Medical School (no. 7511) and financed exclusively from departmental funds.

### Setting and population

The participants were professionals of a university hospital (tertiary referral centre). In the year before the training, 2982 childbirth were recorded, of which 919 were caesarean sections, 54 were emergency caesarean sections. Up to the training, there were training events by means of lectures and separated practical exercises of technical skills. Participation in the questionnaire study was voluntary and could be terminated at any time without giving reasons. The setting has already been described an devaluated in another context with regard to subjective competence gain [16] and transfer of the training content into everyday clinical practice [17]. Participation in the training was planned and specified by the supervisors. In total, 25 theatre nurses, 31 midwives, 23 obstetricians, 46 anaesthetic nurses and 26 anaesthesiologists (in total 151 participants). At last, 80% of the delivery room staff of each department was trained. No other simulation trainings were performed before and during the study.

### Training course

The intervention was scheduled as a 4 h simulation-based emergency caesarean section training in an inter-professional team with all involved professional groups [18]. The training goals were to train the department-specific standard operation procedure of an emergency caesarean section and to improve non-technical skills. In the context of these objectives, it was possible to train in the simulation center, unlike training in process flows [19]. The availability of an audio–video debriefing facility and the possibility of normal clinical care in the delivery room were further arguments. Neonatal care was not included in the scenarios because, in the experience of the trainers, the scenarios become too long and complex. After an introductory lecture, each participant has the opportunity to take part in two scenarios actively and in two scenarios as an observer. The scenarios included the care of the mother in the delivery room, the decision to perform an emergency caesarean section, alerting the operation team, transfer of the patient to the operation room, induction of a general anaesthesia and skin incision. All scenarios were recorded on video and a debriefing according “TeamGAINS” methodology [20] was conducted.

### Questionnaire

#### Presence

The scale “Presence Scale for Lab-based Microworld Research” (PLBMR) was devised by Frank and Kluge [11]. All six items of the questionnaire were rated in a 6-point Likert scale from 1 = “totally disagree” to 6 = “totally agree” (Table 1). The internal consistency of the scale proved to be alpha = 0.71 [11]. The mean of all items is used as a marker for the presence during the simulated scenarios (one item is used reverse-scored).

**Table 1:** Items of Presence Scale for Lab-based Microworld Research (PLBMR) for German and English trainees [11]

German	Translated into English
Introduction: You will now read a series of statements that each describe their perception of the simulation world. Indicate to what extend the statement applies. The size of the number correlates with the approval. Three examples were given with big approval and big dislike. Items could be answered on a 6-point-Likert-scale from 1 (“trifft nicht zu”/ “I totally disagree”) to 6 (“trifft vollständig zu”/ “I totally agree”)	
Ich habe mich als Teil der Simulationswelt gefühlt	I felt like I was part of the Simworld
Die Simulationswelt hat bei mir Emotionen (z.B. Ärger, Traurigkeit, Zufriedenheit) ausgelöst	The simulation world triggered my emotions (e.g. anger, sadness, satisfaction)
Die Arbeit mit der Simulationswelt war für mich zufriedenstellend	Working in the simulation world was satisfying for me
Während ich in der Simulation war, habe ich zwischenzeitlich vergessen, dass ich an einer Studie teilnehme	While operating the simulation, I forgot for the time being that I was taking part in a study
Die Arbeit in der Simulationswelt war für mich langweilig (R)	Working in the simulation world was boring for me
Während ich in der Simulation war, bin ich gedanklich in die Simulationswelt abgetaucht	While operating the simulation, my thoughts became immersed in the simulation world

Items with an (*R*) are negatively worded and have to be reversed-scored

#### Evaluation of the course by the participants

In the work environment of the study participants, it cannot be assumed that the terms around the research of non-technical skills are known. To assess the impact of the training, authors therefore chose terms which, in their experience, are most frequently used by the employees in the context of an emergency caesarean section (6 Items). The questionnaire can be found in the supplement material. The assumed effect of the training should be rated on a Likert-scale of 1 = “strong effect” to 6 = “no effect”. In addition, participants were asked to rate the statement: “I have benefited from the course for my everyday working life” (Likert Scale: 1 = strongly agree to 6 = strongly disagree). To proceed the overall evaluation of the course, the six-point scale of the German school rating system was used, which is considered to be generally known (1 = “very good”–6 = “unsatisfactory”). To assess the participant’s willingness to take part in a future simulation session, a three-point Likert scale was used: 1 = “participate not voluntary” to 3 “gladly participate”.

### Statistical analysis

The demographic and survey data were analysed in a descriptive manner. For testing hypothesis (a), the reliability of the scale, Cronbach’s alpha was calculated. The normal distribution was revised using the Kolmogorov–Smirnov test. Differences in the evaluation by the professional groups (hypothesis b) were examined by the Kruskal–Wallis test.

To test hypothesis c-f, whether the presence correlates with rating and subjective effect of the course, a Kendall-Tau-b correlation was calculated. We assumed a *p* < 0.05 as being statistically significant. The effect size was interpreted according to Cohen as small (> 0.1), medium (0.3–0.5) and high (> 0.5) [21]. All calculations were conducted using SPSS Statistics 26 (IBM Corporation, Forster CITY, CA, USA).

## Results

In total, 106 participants completed the questionnaire (70 percent of the course participants). The data are shown by professional group in Table 2.

**Table 2:** Participants of the inter-professional team training on the topic of emergency caesarean section

Professional group	Trained professionals	Answered questionnaires
Theatre nurse	25	21
Midwife	31	21
Obstetrician	23	14
Anaesthesia nurse	46	24
Anaesthesiologists	26	26

### Reliability of the scale

To test hypothesis (a), Cronbach’s alpha was calculated. The result for the presence questionnaire was 0.72. No item reduction led to a further substantial increase in Cronbach’s alpha.

### Rating and evaluation by professional group

**Fig. 1:**
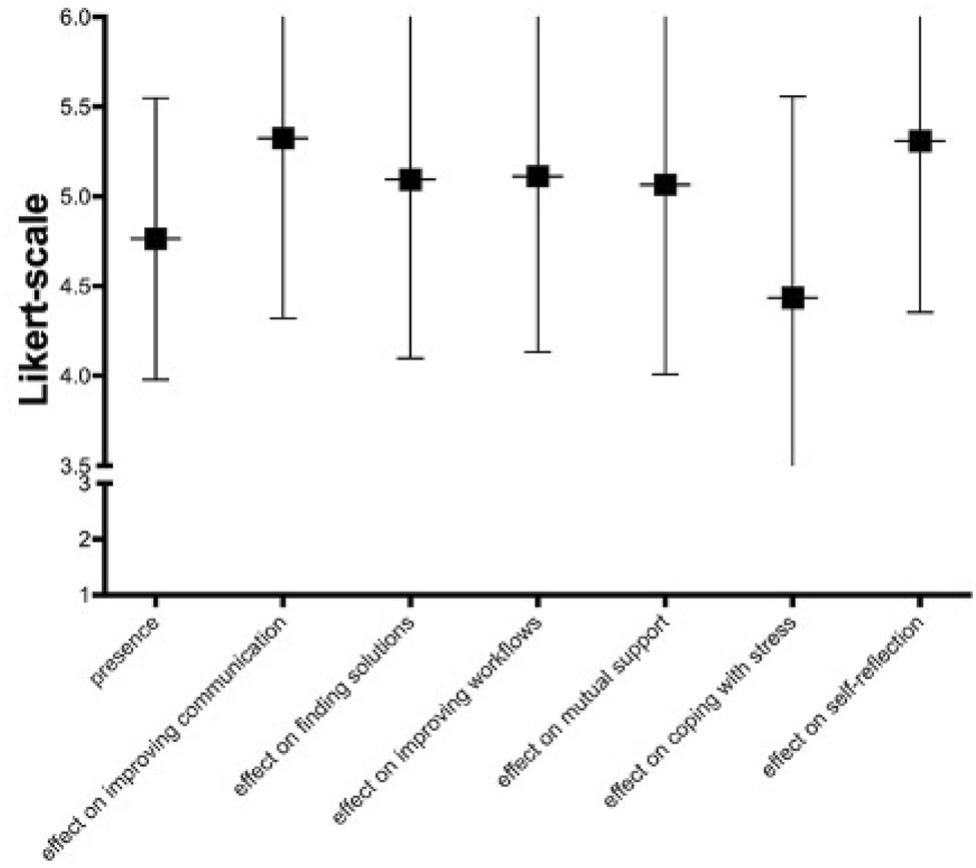
Evaluation of Presence and assumed training objectives of the simulation, an emergency cesarean section (6-Point Likert-scale of 1 = “strongly effect” to 6 = “no effect”). All items are rated higher than 4.4

To evaluate the presence, suspected effects on non-technical skills and attitude of the individual to further training (hypothesis b), the arithmetic mean was calculated within each item. The presence was rated mean = 4.76 (SD = 0.78). Values of the suggested effects are shown in Fig. 1. The benefit for the own daily work was evaluated with mean = 1.63 (SD = 0.98). The overall evaluation was rated with mean = 1.63 (SD = 0.87). The willingness to take part in a repetitive training was rated with mean = 2.66 (SD = 0.51).

The results of the Kolmogorov–Smirnov test showed not normally distributed data. There were no significant differences in the ratings between the professional groups (hypothesis b).

### Correlation

The correlation of presence and effects on non-technical skills is shown in Table 3. (hypothesis c). All questioned items showed a significant correlation, with the parameter “mutual support” achieving a high effect, self-reflection and finding solutions a medium effect. The correlation of presence and evaluation of the course is shown in Table 4. “Overall evaluation of the course” and “willingness to participate in another simulation training” showed a medium effect. All three items correlated significantly with the perceived presence (hypothesis d-f).

**Table 3:** Correlation between level of Presence and subjective effects on non-technical skills and personal attributes (Kendall-tau-b test; *p* value: level of significance *p* < 0.05)

Presence–subjective effect on	Correlation	*P* value
Improving communication	0.196	0.016
Improving finding solutions	0.311	< 0.001
Improving workflows	0.203	0.011
Mutual support	0,639	0.000
Coping with stress	0.202	0.010
Self-reflection	0.335	0.000

**Table 4:** Correlation between level of Presence and evaluation of the course (Kendall-tau-b test; *p* value: level of significance *p* < 0.05)

Presence–evaluation	Correlation	*P* value
Benefit for everyday working life	0.292	< 0.001
Overall evaluation of the course	0.395	< 0.001
Willingness to participate in another simulation training	0.350	< 0.001

## Discussion

The aim of our study was, to test the assessment of presence in the target group of an inter-professional delivery room team and to describe its effects on the subjective perception of the participants.

## Main findings

### Reliability of the presence scale

The value of Cronbach’s alpha was 0.72 which is good for low-stakes instruments [22]. Compared to a previous study (Cronbach’s alpha 0.53 (acceptable)) [15], this is a higher value, which is interesting, because the number of professional groups involved in a delivery room training tend to be more. In the comparative study, only two professional groups, anesthetist and anesthesia nurse were involved in an incident training, but they worked in a wide variety of anesthesia departments [15]. Participants´ history, previous social interaction, and past psychological history are known as influence factors on perceived reality [23].

Here, the work environment of the participants seems to have more influence on the reliability of presence than the professional group. Consequentially, scenarios should be adapted to the special needs of the participants in various departments. For delivery room training, for example, an evaluation with professional groups working in different level of cares might be necessary.

### Different evaluation of the training by professional group

The argument, that the professional group is not decisive is supported by the fact, that in our study, we did not have any significant differences in all assessments by the professional group (hypothesis b). The scenarios of an emergency caesarean section seem to be equally suitable for all professional groups to generate an equivalent presence.

All evaluation items were rated good (all values above 4.4), which was not surprising as the training had already been evaluated in terms of subjective competence gain [16].

### Presence as one success factor for simulation training

The presence correlated significantly with all evaluated items of non-technical skills (hypothesis c). This correlation supports the approach that the perceived reality is correlated to learning as one factor and is in line with this part of the literature [14]. Especially when trainings are ineffective, as discussed in obstetric trainings [6], a lack of presence or similar concepts can provide clues to improve the simulations. This must not be reduced to the use of different simulators—e.g., high versus low fidelity. Furthermore, there are studies that showed the superiority of a low-fidelity simulator [24, 25]. In addition to a physical fidelity, a high psychological fidelity and cognitive fidelity are especially important [26]. To create a high perceived reality of the participants, several conditions have to be met. Availability of expected material, demands for underlying cognition and information processing, disturbing jumps in time and unnatural interaction with the patient are some examples [27]. It is not surprising that the effects were high, as other factors also have influence on the success factors of training courses. In addition to the mentioned debriefing concept, there are additional influence factors like the role of the teacher [28].

## Strengths and limitations

This study hast the limitation of having a monocentric design. Training and safety culture of the individual department could influence the willingness to train and thus also the perception of the simulation. Due to the voluntary nature of the study, not all participants took part in the survey and a pre-selection is possible. However, we consider the response rate almost 70% to be almost acceptable. The professional group of anesthesia nurses shows a lower participation. The training was organized so that the anesthesia nurses of the first course (two trainings a day) replaces the colleagues of the second course in the operation theatre. In our opinion, this time, pressure influences the willingness to participate.

## Interpretation

Since starting our anesthesia-focused studies, there was no dependence of presence on teamwork [15]. Here the highest correlation of presence is with the item “mutual support”, which can be assigned to the non-technical skill “teamwork” [29]. This aspect is likely increased, the more interdisciplinary and inter-professional groups are involved in the training. The scenarios require procedures, which are not necessarily assigned to one medical specialty (e.g., positioning the patient on the operating table). Even if there is a clear allocation in the standard of the clinic, distractors always occur (e.g., different times of arrival of the team members) and need handling. The perceived reality increases the pressure to act for the participants. The scenarios in anesthesia settings are mostly single-placed. There is no challenge of changing the place of action during urgent medical measures.

The item “improving finding solutions” can be interpreted in line with this argumentation. Based on the as-if-concept, who perceived the effects of the clinical rules (here time pressure during emergency cesarean section), these circumstances influence the item.

It is also understandable for the item “self-reflection”. A participant, as experienced professional, only reviews his own behavior when the observed situation feels real. This interpretation follows the theory that adults learn through a process of working out what possible explanations are and sorting them into probable and less probable, on the basis of reflecting on feedback, on existing experience and knowledge [30].

The item “improving communication” has the lowest correlation with the measured presence. This was not expected, as communication is seen as one of the core-elements of non-technical skills [31]. On the Likert Scale of evaluation, the item reaches the highest level, so other success factors of the course must influence the evaluation of communication improvements.

The trained professional groups meet in everyday practice mainly in clinical care. The course offers a rare opportunity to meet with other professional groups in a protected environment. A positive effect of simulation as a method for teambuilding has been described before [32] and, according to our interpretation, is highly valued in the study group for the item “communication”. The training of standard keywords (e.g., anesthesiologist: “Tube is in-incision!”), may not require high levels of scenario reality. These aspects can be noted as training of processes rather than simulation training.

The evaluation of the items “significance for daily work”, “overall evaluation” and “willingness to participate again” (hypothesis d, e, f) can be classified as level one according to Kirkpatrick [33]. The correlation of presence with these items shows that presence is a factor of reaction and should be subsequently valued. Implementation and compliance of standard operation procedures are challenging and needed continuous efforts over time [34]. Participant´s acceptance and appreciation of the training will be important for the long-term implementation of a training program, as postulated by Draycott et al [7].

## Conclusion

The scale of presence is applicable in the professional groups working in a delivery room. The presence correlates with the assumed training objectives and the evaluation of the course. This could be a success factor for the implementation of a continuous training concept. If simulation training is not successful, the perceived reality of participants in the training would be a possible point of improvement. The concept of presence provides an easy-to-use method.

## Data Availability

The datasets generated during and/or analysed during the current study are available from the corresponding author on reasonable request.

## Code availability

All code for data cleaning and analysis associated with the current submission are available from the corresponding author on reasonable request.
